# Effect of the CSFV NS5A protein on key proteins in the MAPK and PI3K-mTOR signaling pathways in porcine macrophages

**DOI:** 10.3389/fmicb.2025.1559840

**Published:** 2025-02-26

**Authors:** Yidan Wang, Xiaoru Zheng, Yingying Yang, Xinru Zhao, Min Li, Juan Huang, Qiaoya Zhang, Xiaobing Qin, Ying Yu, Qing Pan, Zhi Cao

**Affiliations:** College of Veterinary Medicine, Qingdao Agricultural University, Qingdao, China

**Keywords:** CSFV NS5A, lentivirus, MAPK pathway, PI3K-mTOR pathway, differential protein

## Abstract

Classical swine fever (CSF) is a highly contagious disease caused by classical swine fever virus (CSFV). NS5A, a non-structural protein of CSFV, plays an important role in regulating viral replication and protein translation. The purpose of this study was to investigate the effects of the CSFV NS5A protein on key proteins in the mitogen-activated protein kinase (MAPK) and phosphoinositide 3-kinase (PI3K)-mechanistic target of rapamycin (mTOR) pathways in porcine macrophages. In this study, an NS5A lentivirus was constructed, and 3D4/21 cells were infected. The cells infected for 48 h were collected for proteomic analysis to screen the differential proteins in the two signaling pathways in the NS5A/control group, and the expression levels of key proteins were verified by Western blotting (Wb). CSFV NS5A lentivirus was successfully constructed and used to infect porcine macrophages, and 23 upregulated proteins and 16 downregulated proteins were found in the MAPK signaling pathway, whereas 5 upregulated and 15 downregulated proteins were found in the PI3K-mTOR signaling pathway. The results revealed that with increasing infection time, the expression of IKBKG, AKT1, CDC37, MAP3K2, and PKN2 decreased, whereas the expression of MAP3K7 and KRAS2 increased. The 3D4/21 cells infected with NS5A lentivirus and classical swine fever virus were inoculated, and the differential protein expression was verified via Wb. With increasing time, the protein expression levels of IKBKG and KRAS2 increased, whereas the protein expression levels of MAP3K7, MAP3K2, AKT1, CDC37, and PKN2 decreased. This study provides data for revealing the mechanism by which CSFV evades host antiviral immune clearance and has important scientific significance and potential application value.

## 1 Introduction

Classical swine fever (CSF) is a highly contagious infectious disease caused by classical swine fever virus (CSFV). CSF is listed as a legally reported animal disease by the World Organization for Animal Health (WOAH) and as a second-class animal disease in China. CSFV, belonging to *Flaviviridae, Pestivirus*, is a single-stranded positive-sense RNA virus with a genome size of 12.3 kb that encodes a polyprotein (Ganges et al., 2020). The polyprotein is cleaved into four structural proteins (C, E^rns^, E1, and E2) and eight non-structural proteins (N^pro^, P7, NS2, NS3, NS4A, NS4B, NS5A, and NS5B) by proteases (Zhou, 2019). NS5A is a multifunctional phosphorylated protein containing 497 amino acids and is the only replication complex that can complement each other in translation. It participates in CSFV genome replication and achieves effective infection and sustained proliferation in host cells (Risager et al., 2013).

After the virus infects the body, the cell initiates a variety of cellular signaling pathways to establish an antiviral state. MAPK is the main cellular signal transduction pathway. Studies have shown that MAPK interacting kinase 1 (MNK1) regulates cap-dependent and internal ribosome entry site (IRES)-mediated mRNA translation (Li et al., 2023). In the MAPK signaling pathway, cell surface receptors activate the downstream signaling molecule mitogen-activated protein (MAPK) kinase after receiving the signal and then enter the nucleus to phosphorylate a variety of transcription factors and target proteins, such as extracellular signal-regulated kinase (ERK), which is related to cell proliferation differentiation; p38 kinase, which is related to cell stress and apoptosis; and c-Jun N-terminal kinase (JNK) (Kumar et al., 2018). In the PI3K–mTOR signaling pathway, IFN-I binds to the receptor, activates PI3K, and then activates the mTOR complex (Cantley, 2002). The complex can bind to rapamycin target protein (RMT), activate serinase (SP) after interaction with AKT, and activate G protein signal regulator (RGT) after interaction with STAT (Cargnello and Roux, 2011).

After CSFV infection, it activates the IFN-I signaling pathway, promotes the expression of a variety of Interferon-Stimulated Genes (ISGs), and then encodes proteins, such as ISG15, Mx (porcine Mx1, porcine Mx2), GBP1, and pOASL (Xie et al., 2021). The synthesized key proteins have been shown to have anti-CSFV effects (Li et al., 2016). The MAPK and PI3K–mTOR pathways affect ISG transcription and mRNA translation. Studies have shown that NS5A can significantly upregulate STAT1 and STAT5 (Mazewski et al., 2020), downregulate the expression of AKT, and significantly inhibit the phosphorylation of mTOR (Cordek et al., 2011). In the MAPK signaling pathway, MAPK1/3 is activated during CSFV infection and is an upstream regulator that induces autophagy, participating in the autophagy process mediated by CSFV through the MAPK1/3-mTOR pathway (Xie et al., 2021). In the AKT–mTOR branch of the PI3K–mTOR signaling pathway, CSFV infection is activated by a reduction in the level of AKT phosphorylation caused by CSFV infection, which affects the level of autophagy. Moreover, NS5A induces autophagy by activating the CAMKK2-PRKAA-mTOR signaling pathway (Huang et al., 2013). Overall, the AKT–mTOR pathway plays an important role in CSFV-induced autophagy and virus replication. During CSFV infection, the NS5A protein plays a very important role in regulating viral genome replication, protein translation and assembly.

The purpose of this study was to explore the effects of the classical swine fever virus NS5A protein on the MAPK and PI3K-mTOR signaling pathways in porcine macrophages and to identify a new antiviral protein or antiviral target for resistance to CSFV.

## 2 Materials and methods

### 2.1 Antibodies and reagents

The following antibodies were used in this experiment: horseradish peroxidase (HRP)-labeled goat anti-mouse IgG antibody (Cat. No: A2016) and horseradish peroxidase (HRP)-labeled goat anti-rabbit IgG (Cat. No: A0208) purchased from Beyotime Biotechnology (Shanghai, China). The anti-KRAS rabbit mAb (Cat. No: PTM6184), anti-PKN2 rabbit mAb (Cat. No: PTM6538), anti-MAP3K7 mouse mAb (Cat. No: PTM5764), anti-AKT1 rabbit mAb (Cat. No: PTM6071), anti-IKBKG gamma rabbit mAb (Cat. No: PTM6546), anti-Laminin beta 1 rabbit mAb (Cat. No: PTM6660), anti-MAP3K2 rabbit mAb (Cat. No: PTM 20293), and anti-Cdc37 rabbit mAb (Cat. No: PTM 7417) were purchased from Jingjie PTM BioLab (HangZhou) Co., Inc.

### 2.2 Cells

3D4/21 cells were maintained in the Laboratory of Preventive Veterinary Medicine, Qingdao Agricultural University, in RPMI 1640 medium supplemented with 1% penicillin–streptomycin (purchased from HyClone Company, China). 3D4/21 cells were cultured in RPMI 1640 medium containing 10% fetal bovine serum (purchased from Vazyme Biotech Co., Ltd.) and 1% penicillin–streptomycin at 37°C and 5% CO_2_.

### 2.3 Lentivirus packaging and infection

The primers used for lentivirus and infection were the public CSFV NS5A sequence on “NCBI” and the chronic disease virus overexpression vector “pCDH-CMV-MCS-EF1 Lentivector Series.” NS5A-specific primers were designed with Primer Premier 5, which was synthesized by Sangon Biotech (Shanghai) Co., Ltd. *Escherichia coli* DH5α competent cells were purchased from Vazyme Biotech (Nanjing) Co., Ltd. PGEX-6P-1-CSFV NS5A plasmids, human embryonic kidney 293T cells (HEK-293T), pCDH-CMV-MCS-EF1 Lentivector Series plasmids and CSFV NS5A proteins were maintained in the Laboratory of Preventive Veterinary Medicine, Qingdao Agricultural University.

In this study, we used three plasmid packaging systems, including the plenti-GIII-CMV-CBH-GFP-2A-Puro-CSFV NS5A transfer plasmid, the pSPAX2 packaging plasmid, and the envelope plasmid pMD2G. HEK-293T cells infected with CSFV NS5A were cultured in a cell incubator. The collected and packaged virus particles were lysed on ice and centrifuged at 25,000 rpm for 1.5 h. The supernatant was discarded, and the virus particles were resuspended in precooled PBS and stored at −80°C.

The packaged CSFV NS5A lentivirus was used to infect HEK-293T cells in a 96-well plate. The cell density in each well was ~1 × 10^6^ cells/mL. Samples were collected at 24 h postinfection, and Western blotting was performed to verify whether the NS5A protein was successfully overexpressed.

The packaged lentivirus was used to infect 3D4/21 cells in a 96-well plate at a density of ~1 × 10^6^ cells/mL per well. The amount of virus required for different MOIs was calculated according to the cell density and formula, and 10^8^ control lentiviruses were added to a 96-well plate. The best GFP concentration was recorded according to the percentage of target cells expressing the MOI and the degree of cytotoxicity. The best MOI formula was as follows: MOI = lentivirus titer (IU/mL) × added virus volume (mL)/total number of cells to be stained.

The 3D4/21 cells were spread in a 6-well plate at a density of 1 × 10^6^ cells/mL. After 18 h, the cell density reached ~80%. All lentiviruses used were melted at 4°C and stored at 4°C until use. The mixture was replaced with 2% FBS-containing DMEM, lentivirus was added at the optimal MOI, and the mixture was incubated in a cell incubator overnight. Three groups were established.

### 2.4 Proteomic analysis

The cells infected with NS5A lentivirus or GFP control lentivirus for 48 h were washed twice with precooled PBS and RIPA lysis buffer (Cat. No: P0013B; Beyotime, Shanghai, China). A total of 100:1 of phosphorylation inhibitor (Cat. No: ST506; Beyotime, Shanghai, China) was added, 200 μL was added to each well, the mixture was shaken for 1–2 min, the cells were scraped off with a scraper, the cells were suspended in PBS, the mixture was collected in a 1.5-mL centrifuge tube, the mixture was shaken for 15 min on ice, the mixture was centrifuged for 5 min at 4°C and 12,000 × g, and the supernatant was transferred to a new centrifuge tube. The protein concentration of each sample was determined with a BCA protein determination kit. The protein samples were subjected to enzymatic hydrolysis, as shown in Table 1. The liquid chromatographic parameters and gradient elution conditions used for reverse chromatographic separation are shown in Tables 2, 3.

**Table 1:** Protease digestion parameters.

**Reagents**	**Reaction condition**
5 mM Dithiothreitol	56°C, 30 min, cool to room temperature
10 mM Iodoacetamide	Without light for 15 min
200 mM TEAB 1 mg/mL trypsin-TPCK	37°C, 12 h
1 mg/mL Trypsin	37°C, 4 h

**Table 2:** Liquid chromatographic parameters.

**Liquid chromatography**	**EASY-nLC1200**
Mobile phase A	0.1% formic acid and 2% acetonitrile aqueous solution
Detection wavelength	UV 210 nm and 280 nm
Flow rate	500 nL/min
Mobile phase B	0.1% formic acid and 90% acetonitrile aqueous solution
Chromatographic column	Orbitrap Exploris 480

**Table 3:** Gradient elution conditions.

**% B (liquid phase gradient)**	**Time**
6–23	0–68 min
23–32	68–82 min
32–80	82–86 min
80	86–90 min

The data were analyzed via Proteome Discoverer 2.4.1.15, mainly to analyse the interactions among trusted and differential proteins.

### 2.5 Western blot verification of the expression trends of key proteins

3D4/21 cells were seeded in a 6-well plate at a density of 1.0 × 10^6^ cells/mL. After the cell density reached 90%, according to the optimal MOI, NS5A-overexpressing lentivirus and control lentivirus-infected cells were added 24, 48, 60, and 72 h after NS5A lentivirus infection and 24, 48, 60, and 72 h after control lentivirus infection. Blank control cells were collected, and the process was repeated three times. The cell lysate, precooled in an ice bath (containing protease inhibitors), was added and incubated on ice for 30 min. The supernatant was collected by centrifugation at 13,000 × g, and the expression levels of key proteins were detected via Western blotting.

### 2.6 Western blot verification of the impact of the NS5A protein on differentially expressed proteins in porcine macrophages during CSFV infection

To verify the effects of the NS5A protein on the MAPK and PI3K-mTOR signaling pathways in porcine macrophages during classical swine fever virus infection, 3D4/21 cells were plated in a 6-well plate at a density of 1.0 × 10^6^ cells/mL. When the cell density reached ~90%, the packaged NS5A lentivirus was used to infect 3D4/21 cells at an MOI of 500 for 24 h, followed by the addition of CSFV. Samples were collected at 24, 48, 60, and 72 h post-CSFV infection. The cells not infected with CSFV served as the control group, and each group was replicated three times. The cells were washed twice with precooled PBS, precooled cell lysis buffer (containing protease inhibitors) was added, and the mixture was incubated on ice for 30 min before being centrifuged at 13,000 × g to collect the supernatant. The expression trends of the aforementioned seven differential proteins were detected.

## 3 Results

### 3.1 Packaging and infection of lentivirus

**Figure 1:**
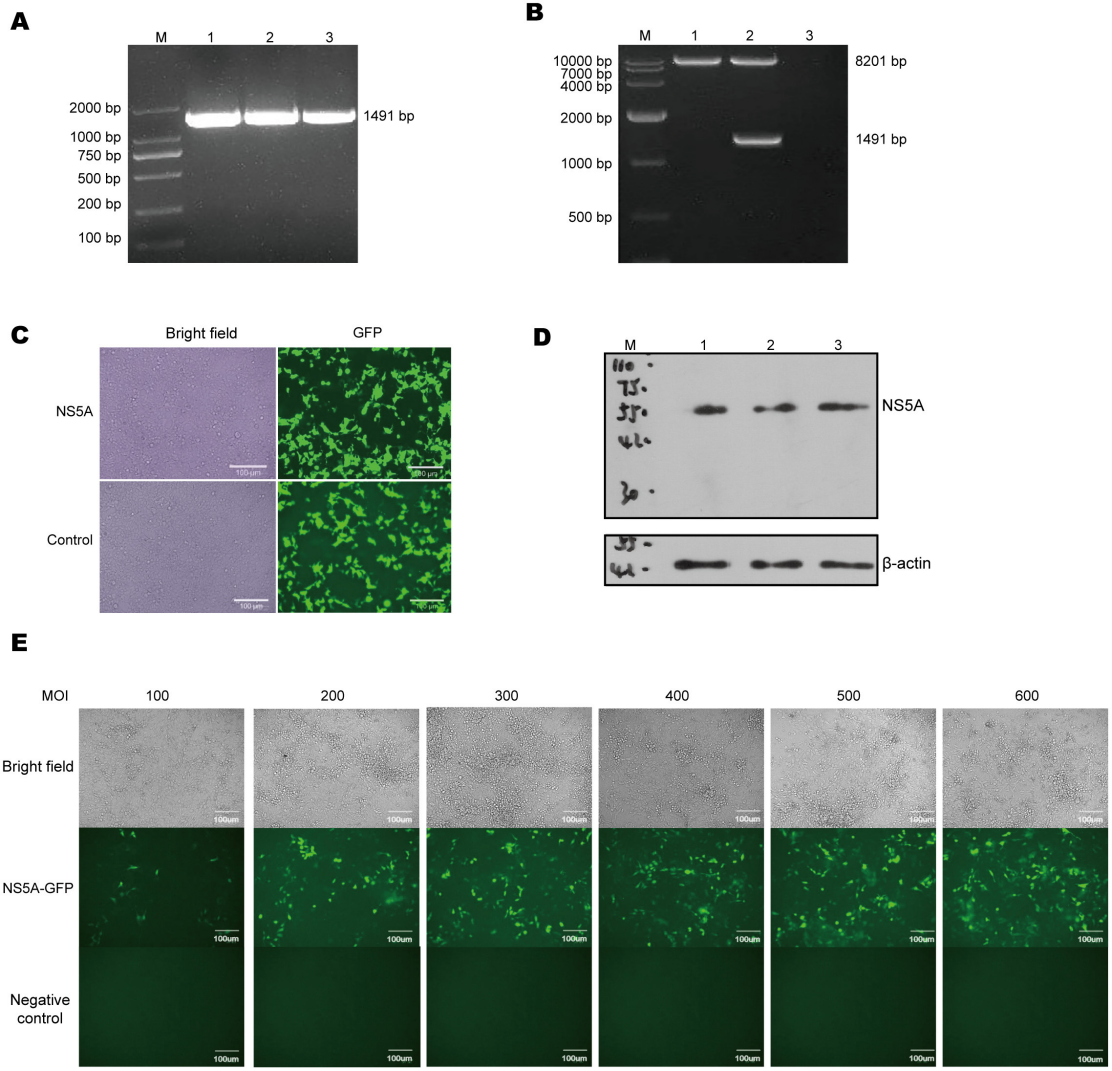
Lentiviral packaging and cell infection. **(A)** Using the full-length CSFV plasmid as a template, the target fragment of NS5A was amplified by PCR, and the size of NS5A was 1,491 bp, among which M: DL 2000 Marker; 1, 2, 3: NS5A. **(B)** After double enzyme digestion of CSFV NS5A lentivirus, the correct size of the target band was obtained, including M: DL 10000 Maker; 1, 2, 3: empty vector lentivirus, CSFV NS5A lentiviral vector and blank control. **(C)** The state and fluorescence of HEK-293T cells were observed 24 h after they were infected with CSFV NS5A lentivirus. **(D)** Western blotting was used to verify whether the packaged CSFV NS5A lentivirus was correctly expressed. M: 150 kDa marker; 1, 2, 3: HEK-293T cells infected with the NS5A lentivirus. **(E)** 3D4/21 cells were infected with CSFV NS5A lentivirus at MOIs of 100, 200, 300, 400, 500, and 600. The best MOI of 500 was obtained by observing the cell state and fluorescence under a microscope. “Negative control” represents the fluorescence condition of cells that have not been infected with the lentivirus; “NS5A—GFP” represents the fluorescence situation of cells infected with the NS5A lentivirus containing the GFP tag.

To achieve long-term stable expression of the target gene in host cells, a CSFV NS5A lentivirus was constructed. The full-length CSFV plasmid was used as the template, and the designed upstream and downstream primers were used to amplify the target fragment, which was 1,491 bp in size (Figure 1A). The CSFV NS5A lentivirus obtained by the three-plasmid packaging system was identified after double enzyme digestion, which revealed that the 8,201 bp and 1,491 bp fragments presented the same and clear bands as expected and that there were no other non-specific bands (Figure 1B). Twenty-four hours after the transfection of CSFV NS5A lentivirus into HEK-293T cells, many green fluorescent spots and cytopathic changes, accompanied by floating and ruptured cells, were observed under a fluorescence microscope. The infection efficiency was >80% (Figure 1C). The cell samples infected with the lentivirus were collected. The Western blotting results revealed a distinct band at 50 kDa, indicating that the NS5A lentivirus was successfully overexpressed (Figure 1D). In a 96-well plate, 3D4/21 cells were infected with successfully packaged CSFV NS5A lentivirus. At MOIs of 100, 200, 300, 400, 500, and 600 for 24 h, the optimal MOI was determined according to the cell state and fluorescence. The optimal MOI of 500 was obtained on the basis of infection efficiency, the cell survival rate and the cell damage rate (Figure 1E).

### 3.2 Proteomic analysis was used to assess changes in total protein expression levels

**Figure 2:**
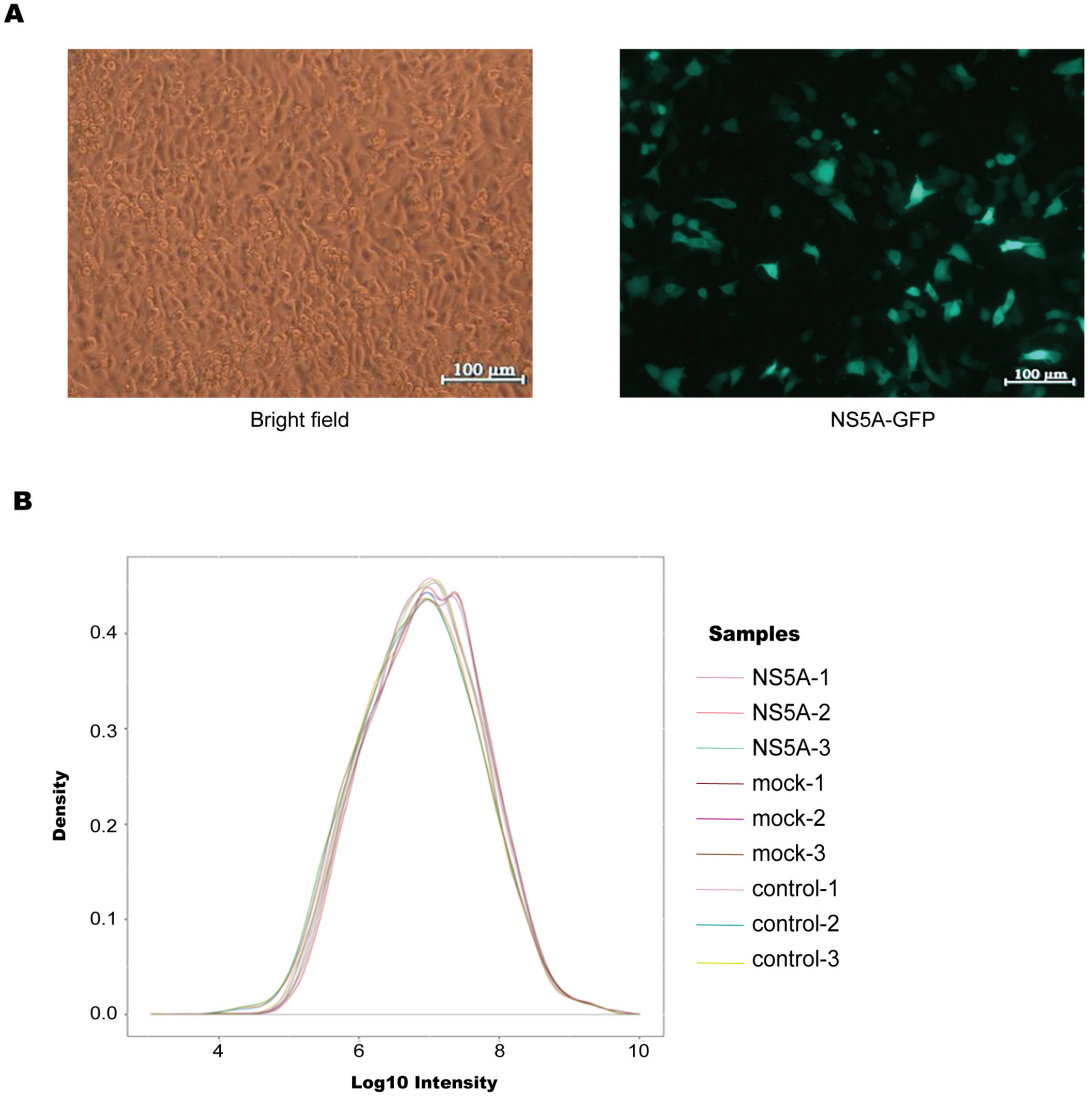
Overall proteomic analysis. **(A)** According to the optimal MOI of 500 for the infection of 3D4/21 cells, the infection was successful, and the cells were in good condition. **(B)** Density distribution map, which directly reflects the distribution of protein intensity values in each sample. The horizontal axis represents the intensity value after Log10 conversion, the vertical axis represents its probability density, the peak of the density distribution curve indicates that the number of proteins at this level was the greatest, and the protein strength between groups tended to increase, indicating that the reliability of egg white analysis was high.

To explore how NS5A regulates virus replication, the successfully packaged lentivirus was infected into 3D4/21 cells (Figure 2A) at the most suitable MOI of 500. The samples were collected, and the protein content was determined via the BCA method. The results revealed that the sample table was good, the concentration was normal, and the amount of residual protein was sufficient (Table 4). According to the density distribution map of the intensity values of all the sample proteins, the number of proteins was greatest when the log10 value was in the range of 7–7.5, and the protein intensity between groups tended to aggregate, indicating that the reliability of the protein analysis was high (Figure 2B).

**Table 4:** Determination of sample concentration.

**Serial number**	**Sample number**	**Protein concentration (μg/μL)**	**Total sample volume (μL)**	**Total protein (μg)**
1	NS5A (1)	2.83	33.0	93.39
2	NS5A (2)	2.85	33.0	94.05
3	NS5A (3)	2.81	33.0	92.73
4	Mock (1)	1.04	70.0	72.92
5	Mock (2)	1.05	70.0	73.49
6	Mock (3)	1.08	70.0	75.51
7	Control (1)	1.59	25.0	39.75
8	Control (2)	1.62	25.0	40.50
9	Control (3)	1.58	25.0	39.50

These results indicate the high confidence and repeatability of the proteomic analysis data.

### 3.3 Identification of differentially expressed proteins

**Figure 3:**
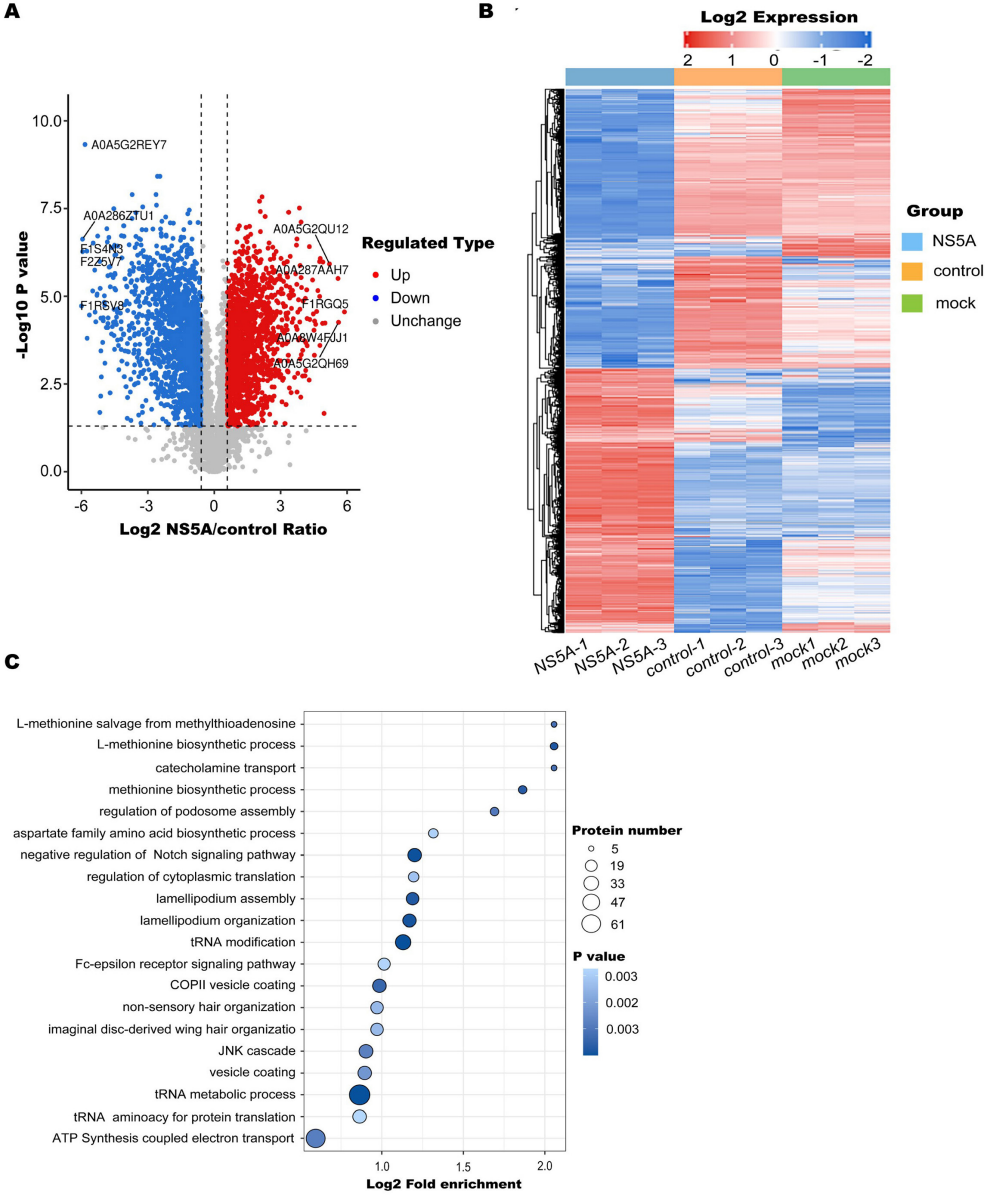
Differentially expressed protein enrichment analysis. **(A)** Volcano diagram. The horizontal axis represents the value of the differential expression change ratio of the comparison group after Log2 conversion, and the vertical axis represents the statistical test value of the *t*-test *P*-value after Log10 conversion, in which the red dots indicate significant differences in gene expression, the blue dots indicate significant differences in gene expression, and the gray dots indicate no significant differences. **(B)** A heatmap was used to show the relative expression levels of multiple differentially expressed proteins in different samples, revealing the clustering relationship of the relative expression of different proteins. There was one differential protein for each behavior, and each was listed as a sample. Red represents high expression, blue represents low expression, and gray represents an indefinite quantity in the corresponding sample. **(C)** GO enrichment analysis was performed on the differentially expressed proteins of the NS5A/control group. The significance *P*-value was calculated using Fisher's exact test to determine whether the differentially expressed proteins tended to be significantly enriched in certain functional types. The vertical axis of the enrichment bubble chart shows the Gene Ontology (GO) functional description information, and the horizontal axis shows the degree of functional enrichment after Log2 conversion. The higher the value is, the greater the degree of enrichment. The color of the dots represents the *P*-value of enrichment significance. The bluer the color is, the stronger the enrichment significance. The size of the dots represents the number of differential proteins involved in the GO function. The larger the dots are, the greater the number of differential proteins.

To screen for differentially expressed proteins after NS5A overexpression, following the screening and filtering criteria, 3,195 differentially expressed proteins, including 1,539 upregulated proteins and 1,656 downregulated proteins, were identified in the NS5A/control group (Figure 3A). The heatmap shows the relative expression levels of multiple differentially expressed proteins in different samples (Figure 3B). To determine which biological functions were significantly related to the differentially expressed proteins, GO enrichment analysis was used to determine the main biological functions of the differentially expressed proteins, such as those affecting tRNA metabolism, tRNA modification, cytoplasmic translation, and the regulation of actin polymerization or depolymerization (Figure 3C).

### 3.4 MAPK and PI3K-mTOR pathway analysis of differentially expressed proteins

**Figure 4:**
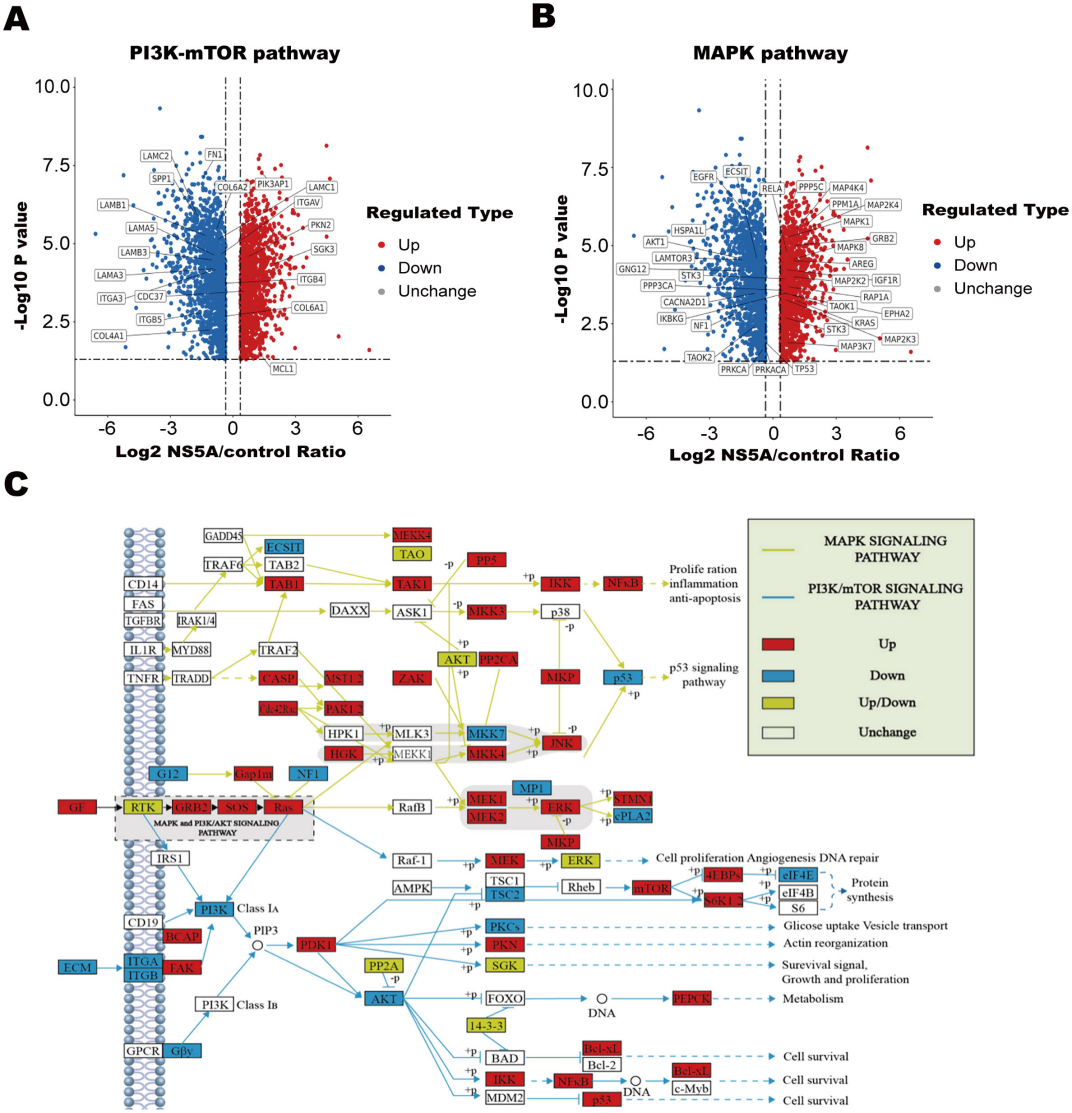
Analysis of differential proteins in the MAPK and PI3K-mTOR pathways and path map. **(A, B)** Volcanic map. The horizontal axis represents the value of the differential expression change ratio of the comparison group after Log2 conversion, and the vertical axis represents the statistical test value of the *t*-test *P*-value after Log10 conversion, in which the red dots indicate that the significant difference was upregulated, the blue dots indicate that the significant difference was downregulated, and the gray dots indicate that there was no significant difference. **(C)** Red filling indicates a differentially upregulated protein, blue filling indicates a differentially downregulated protein, yellow filling indicates that this node contains both a differentially upregulated protein and a differentially downregulated protein, no filling color indicates no significant regulation, the yellow line segment represents the MAPK signaling pathway, and the blue line segment represents the PI3K–mTOR signaling pathway.

To clarify the mechanism of CSFV NS5A in the MAPK and PI3K-mTOR signaling pathways, 23 NS5A/control differential proteins whose expression was upregulated and 16 whose expression was downregulated in the MAPK signaling pathway were screened (Table 5; Figure 4A). All of these proteins play important roles in cell proliferation; for example, MAP3K7 mediates signal transduction induced by TGF-β and morphogenetic protein (BMP) and controls transcriptional regulation and apoptosis. IKBKG helps to activate NF-κB, thus protecting cells from apoptosis induced by TNF-α. The protein encoded by the AKT1 gene is a ubiquitin-like protein that is coupled to intracellular target proteins after the activation of interferon-α and interferon-β. In the PI3K–mTOR signaling pathway, 5 proteins were upregulated, and 15 proteins were downregulated (Table 6; Figure 4B). For example, PKN2 activates RNA polymerase and protein serine/threonine kinase activity, which is related to the positive regulation of viral genome replication; SPP1 is a multifunctional glycoprotein related to active clearance, chemotaxis and macrophage migration of apoptotic cells; and FN1 plays a central role in cell adhesion, regulating cell polarity, differentiation and growth. LAMB1 and LAMC2 are the main non-collagen components of the basement membrane and are involved in cell adhesion, differentiation, migration, signal transduction, neurite growth, and metastasis. According to the differential proteins identified from the two pathways, the corresponding signaling pathway map was drawn to clarify its regulatory trend, which is convenient for verifying its expression in the later stage (Figure 4C).

**Table 5:** Differentially expressed proteins in the MAPK pathway.

**Pathway**	**Characteristic**	**Number**	**Protein name**	**Gene**	**NCBI protein accession no**.		**Max identity**	**NS5A/control**	**NS5A/control**
					**Human**	**Pig**		***P*** **-value**	**Ratio**
MAPK	Up-regulated protein	1	Insulin like growth factor 1 receptor	IGF1R	NC_000015.10	NC_010443.5	99.4%	8.458110	2.054764
		2	Growth factor receptor bound protein 2	GRB2	NC_000017.11	NC_010454.4	98.4%	0.000100	1.951057
		3	Mitogen-activated protein kinase kinase kinase 7	MAP3K7	NC_000006.12	NC_010443.5	99.1%	0.000978	2.848945
		4	TNF receptor-associated factor	TRAF2	NC_000009.12	NW_018084833.1	99.8%	0.000028	1.984416
		5	Receptor protein-tyrosine kinase	EPHA2	NC_000001.11	NC_010448.4	95.1%	0.000247	1.826395
		6	Cellular tumor antigen p53	TP53	NC_000017.11	NC_010454.4	99.7%	0.036582	1.640804
		7	Mitogen-activated protein kinase	MAPK1	NC_000022.11	NC_010456.5	98.3%	0.000500	1.808403
		8	Protein-serine/threonine phosphatase	PPM1A	NC_000014.9	NC_010443.5	88.8%	0.000591	1.677377
		9	Small monomeric GTPase	RAP1A	NC_000001.11	NC_010446.5	93.4%	0.000608	1.802699
		10	GTPase KRas isoform	KRAS	NC_000012.12	NC_010447.5	99.3%	0.000111	4.459623
		11	Heat shock 70 kDa protein 1B	HSPA1B	NC_000083.7	NC_010449.5	97.5%	0.000765	0.613020
		12	Stress-activated protein kinase	MAPK8	NC_000007.14	NC_010449.5	99.4%	0.005336	1.763117
		13	Serine/threonine-protein phosphatase	PPP5C	NC_000019.10	NC_010448.4	94.3%	0.000001	1.420503
		14	Serine/threonine-protein kinase	TAOK1	NC_000017.11	NC_010454.4	92.3%	0.016830	1.621804
		15	Non-specific serine/threonine protein kinase	STK3	NC_000008.11	NC_010446.5	92.8%	0.000442	1.623412
		16	Amphiregulin	AREG	NC_000004.12	NC_010450.4	98.6%	0.000001	1.328570
		17	Mitogen-activated protein kinase kinase	MAP2K2	NC_000019.10	NC_010444.4	96.8%	0.000128	1.736383
		18	Dual specificity mitogen-activated protein kinase kinase 4 isoform 1	MAP2K4	NC_000019.10	NC_010444.4	99.6%	0.000008	1.854659
		19	Serine/threonine-protein phosphatase	PPP3CA	NC_000004.12	NC_010450.4	90.8%	0.000356	1.563399
		20	RELA proto-onco, NF-kB subunit	RELA	NC_000011.10	NC_010444.4	98.2%	0.000007	1.523190
		21	Mitogen-activated protein kinase kinase kinase kinase 4	MAP4K4	NC_000002.12	NC_010445.4	91.4%	0.000024	1.908520
		22	Inhibitor of nuclear factor kappa B kinase regulatory subunit gamma	IKBKG	NC_000023.11	NC_010461.5	99.8%	0.008597	2.757278
		23	Mitogen-activated protein kinase kinase 3	MAP3K2	NC_000017.11	NC_010454.4	97.7%	0.000116	2.802919
	Down-regulated protein	1	Protein kinase C	PRKCA	NC_000017.11	NC_010454.4	92.0%	0.026239	0.546647
		2	Mitogen-activated protein kinase	MAPK3	NC_000016.10	NC_010445.4	98.6%	0.000622	0.551025
		3	Receptor protein-tyrosine kinase	EGFR	NC_000010.11	NC_010456.5	99.7%	0.000000	0.181691
		4	Non-specific serine/threonine protein kinase	RPS6KA3	NC_000023.11	NC_010461.5	98.3%	0.008219	0.666294
		5	Heat shock 70 kDa protein 1-like	HSPA1L	NC_000006.12	NC_010449.5	93.1%	0.000002	0.036804
		6	Calcium voltage-gated channel auxiliary subunit alpha2delta 1	CACNA2D1	NC_000071.7	NC_010451.4	75.5%	0.000023	0.422307
		7	Mitogen-activated protein kinase kinase kinase 2	MAP3K2	NC_000002.12	NC_010457.5	92.5%	0.038008	0.550393
		8	cAMP-dependent protein kinase	PRKACA	NC_000019.10	NC_010444.4	98.5%	0.005365	0.548717
		9	RAC-alpha serine/threonine-protein kinase	AKT1	NC_000014.9	NW_018084979.1	98.4%	0.000035	0.204580
		10	Neurofibromin 1	NF1	NC_000017.11	NC_010454.4	99.3%	0.000038	0.258996
		11	Evolutionarily conserved signaling intermediate in toll pathway, mitochondrial	ECSIT	NC_000019.10	NC_010444.4	98.2%	0.000301	0.491548
		12	Profilin	PFN2	NC_000003.12	NC_010455.5	87.5%	0.000034	0.578601
		13	Protein-serine/threonine phosphatase	PPM1B	NC_000002.12	NC_010445.4	91.9%	0.002238	0.375331
		14	Guanine nucleotide-binding protein subunit gamma	GNG12	NC_000001.11	NC_010448.4	82.6%	0.000012	0.142335
		15	Regulator complex protein LAMTOR3	LAMTOR3	NC_000004.12	NC_010450.4	99.9%	0.000044	0.209502
		16	Serine/threonine-protein kinase TAO2	TAOK2	NC_000016.10	NC_010445.4	77.7%	0.010152	0.488289

**Table 6:** Differentially expressed proteins in the PI3K–mTOR pathway.

**Pathway**	**Characteristic**	**Number**	**Protein name**	**Gene**	**NCBl protein accession no**.		**Max identity**	**NS5A/control**	**NS5A/control**
					**Human**	**Pig**		***P-*** **value**	**Ratio**
PI3K-mTOR	Up-regulated protein	1	Induced myeloid leukemia cell differentiation protein Mcl-1	MCL1	NC_000001.11	NC_010446.5	98.7%	0.032906	1.503720
		2	Hsp90 cochaperone Cdc37	CDC37	NC_000019.10	NC_010444.4	98.8%	0.000327	2.142728
		3	Phosphoinositide-3-kinase adaptor protein 1	PIK3AP1	NC_000010.11	NC_010456.5	85.5%	0.000001	1.077216
		4	Serum/glucocorticoid regulated kinase family member 3	SGK3	NC_000008.11	NC_010446.5	96.6%	0.003542	1.758767
		5	Protein kinase C	PKN2	NC_000001.11	NC_010446.5	98.9%	0.000017	2.156021
	Down-regulated protein	1	Integrin subunit alpha 3	ITGA3	NC_000017.11	NC_010454.4	91.5%	0.000062	0.432642
		2	Integrin beta	ITGB5	NC_000003.12	NC_010455.5	99.2%	0.003121	0.213663
		3	Integrin subunit alpha V	ITGAV	NC_000002.12	NC_010457.5	99.9%	0.000048	0.533435
		4	Osteopontin	SPP1	NC_000004.12	NC_010450.4	98.3%	0.000005	0.113908
		5	Fibronectin	FN1	NC_000002.12	NC_010457.5	99.8%	0.000000	0.178989
		6	Collagen type IV alpha 1 chain	COL4A1	NC_000013.11	NC_010453.5	99.7%	0.000011	0.204592
		7	Laminin subunit beta 1	LAMB1	NC_000007.14	NC_010451.4	99.8%	0.000005	0.191009
		8	Laminin subunit alpha 3	LAMA3	NC_000018.10	NC_010448.4	95.8%	0.000032	0.282988
		9	Laminin subunit alpha 5	LAMA5	NC_000020.11	NC_010459.5	95.7%	0.000000	0.254614
		10	Laminin subunit gamma 2	LAMC2	NC_000001.11	NC_010451.4	92.4%	0.000001	0.126428
		11	Laminin subunit beta 3	LAMB3	NC_000001.11	NC_010451.4	89.8%	0.000002	0.242144
		12	Integrin beta	ITGB4	NC_000017.11	NC_010454.4	92.2%	0.000097	0.546842
		13	Collagen type VI alpha 1 chain	COL6A1	NC_000021.9	NC_010455.5	96.9%	0.001145	0.594640
		14	Laminin subunit gamma 1	LAMC1	NC_000001.11	NC_010451.4	99.8%	0.000002	0.450460
		15	Collagen type VI alpha 2 chain	COL6A2	NC_000021.9	NW_018085356.1	96.9%	0.013060	0.637307

### 3.5 Differential protein expression and interaction

**Figure 5:**
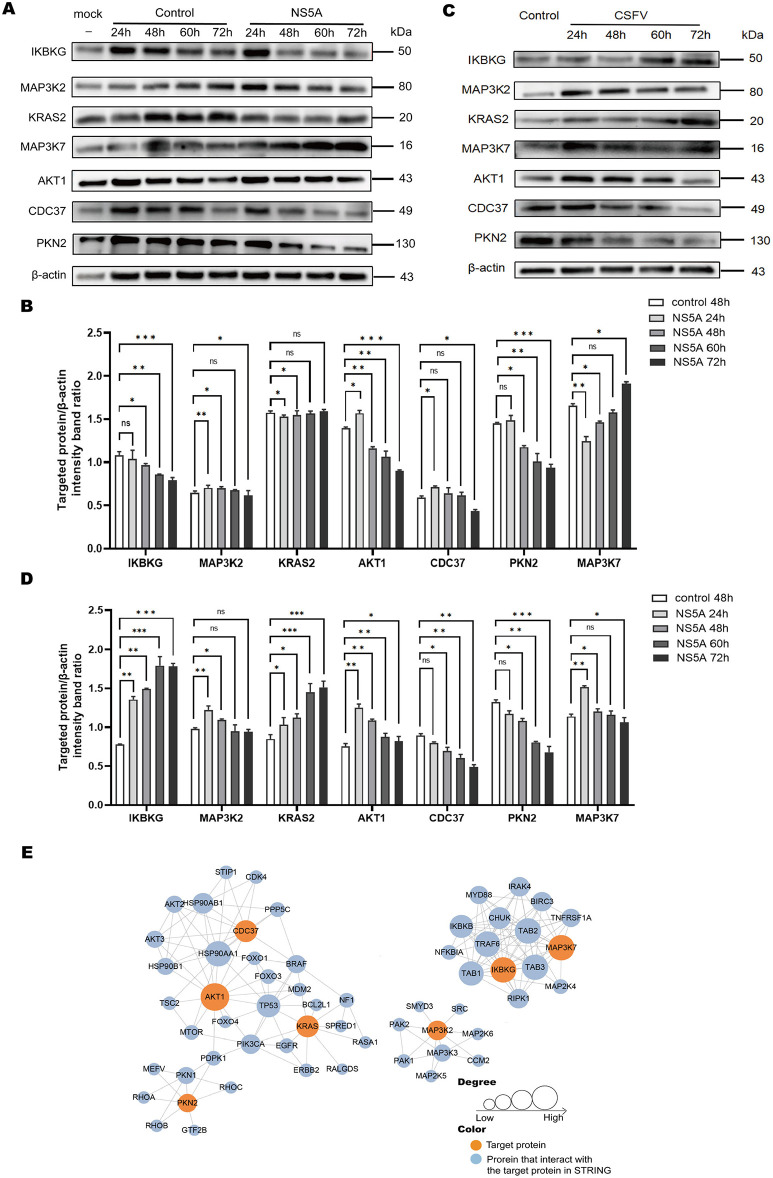
The expression of differential proteins and PPIs. **(A)** Seven key proteins were screened according to a ratio > 2.5 and a *P*-value < 0.05. The 3D4/21 cells infected with CSFV NS5A lentivirus at 24, 48, 60, and 72 h and the empty vector control lentivirus at 24, 48, 60, 72 h and the blank at 48 h were collected to verify the expression of the 7 key proteins over time. **(B)** Cells infected with CSFV NS5A lentivirus at different time points were compared with cells infected with empty vector control lentivirus for 48 h. The data are presented as the mean ± standard deviation (SD) of values from three independent experiments. Significant differences compared with the infection group are expressed as *P < 0.05, **P < 0.01, ***P < 0.001, and ns, not significant. **(C)** “Control” refers to 3D4/21 cells infected with the NS5A lentivirus, while “NS5A 24, 48, 60, 72 h” refers to 3D4/21 cells expressing the NS5A lentivirus after CSFV infection. **(D)** Comparison of 3D4/21 cells expressing the NS5A lentivirus after CSFV infection with cells not infected with CSFV. **(E)** The protein–protein interaction network map of the differentially expressed genes. Orange filling represents the 7 key proteins. After 7 key proteins were compared with the STRING protein interaction network database, the proteins that interact with them were extracted according to a confidence score > 0.7 (high confidence) and filled with blue. The larger the circle is, the stronger the interaction.

To determine whether NS5A affects the expression of differential proteins, according to the principle that “the smaller the *P*-value and the larger the ratio, the more significant the difference,” proteins with *P* < 0.001 and a ratio > 2 were selected, and Western blotting was used to detect the changing trends in the expression of differential proteins at different time points. The results revealed that the AKT1, IKBKG, CDC37, MAP3K2, and PKN2 proteins were decreased, whereas the KRAS2 and MAP3K7 proteins were increased (Figures 5A, B). To verify the impact of the NS5A protein on the MAPK and PI3K-mTOR signaling pathways in porcine macrophages under CSFV infection conditions, CSFV was used to infect 3D4/21 cells expressing NS5A lentivirus, and Western blotting was used to detect the protein expression levels in the MAPK and PI3K-mTOR signaling pathways. Under CSFV infection conditions, the NS5A protein affects proteins in the MAPK and PI3K-mTOR signaling pathways in porcine macrophages. With increasing infection time, the expression levels of IKBKG and KRAS2 increased, whereas the expression levels of MAP3K7, MAP3K2, AKT1, CDC37, and PKN2 decreased (Figures 5C, D). The differentially expressed protein interaction network revealed that the proteins with strong interactions were involved mainly in cell differentiation and signal transduction (Figure 5E).

## 4 Discussion

*Flaviridae* viruses have a variety of immune escape strategies (Xie et al., 2021). To date, many inhibitory compounds that target host metabolism and posttranslational modifications, aimed at interfering with viral replication and the function of viral proteins, have been studied. These compounds exert antiviral effects by targeting the enzyme activities of viral non-structural proteins, especially NS5 methyltransferase (MTase) and RNA-dependent RNA polymerase (RdRp) (Huang et al., 2013). The Wuhan Institute of Virology, CAS, made a breakthrough in the study of the Japanese encephalitis (JEV) non-structural protein NS5, analyzed the crystal structure of Japanese encephalitis virus NS5, and clarified the conservative intramolecular interaction between MTase and RdRP (Huang et al., 2013; Lu and Gong, 2013). Two forms of phosphorylated p56 and p58 of NS5A play important roles in the life cycle of HCV (Slon Campos et al., 2018). NS5A forms a replication complex with HCV RNA and NS5B to complete HCV replication (Chen et al., 2018).

Extracellular signal-regulated kinase (ERK, also known as p42 MAPK), Janus kinase [JNK, also known as stress-activated protein kinase-1 (SAPK1)] and p38 MAP kinase (also known as SAPK2/RK) are the three main MAPK pathways in mammals (Roux and Blenis, 2004). Studies have shown that the MAPK-ERK pathway plays an important role in the regulation of HCV replication and protein expression. MAPK-ERK pathway inhibitors increase the HCV RNA load and NS5A protein content in Con-1 cells and promote translation guided by ribosome entry sites in HCV (Slon Campos et al., 2018). The HCV NS5A protein interferes with the activation of the MAPK-ERK pathway by altering the transport of epithelial growth factor (EGF) receptors, thus weakening the cellular response to EGF (Macdonald and Harris, 2004). Bicyclol inhibits the activation of the MAPK pathway induced by HCV by reducing the level of cellular peroxide, resulting in a decrease in the level of pNF-κB (Gaur et al., 2011; Gretton et al., 2010). The PI3K–mTOR pathway affects the life cycle of HCV by regulating a variety of biological processes in host cells, including protein synthesis, cell growth, proliferation and survival (Pei et al., 2012). For example, the NS5A protein can stimulate PI4KIIIα to produce the phosphatidylinositol 4-phosphate (PI4P) necessary for HCV replication (Macdonald et al., 2005). PI4P is an important part of the cell membrane and can provide a suitable environment for viral replication (Li et al., 2018).

The classical swine fever virus NS5A protein can regulate a variety of biological processes and plays an important role in CSFV growth and virus RNA synthesis (Glaviano et al., 2023). Zhang and others have shown that NS5A is the main functional protein involved in CSFV-induced mitochondrial autophagy, which promotes the replication and proliferation of CSFV (Siu et al., 2016). NS5A reportedly activates autophagy through the PP2A-DAPK3-Beclin 1 axis (Chen et al., 2012). The CSFV NS5A protein can interact with many host proteins. Studies have shown that the NS5A protein interacts with eukaryotic translation initiation factor 3 subunit E (eIF3E), Ras-associated protein 18 (Rab-18), glucose-regulated protein 78 (GRP78) and heat shock protein 70 (Hsp70) to promote viral replication (Chengcheng et al., 2022; Zhang et al., 2020).

Lentiviral vectors can effectively integrate foreign genes into the genome of host cells, achieve long-term stable expression, and have the advantage of persistent expression of the target sequence (Zhang et al., 2023; Chengcheng et al., 2020). The study of host cell proteins is the most extensive (Ku et al., 2021). Proteomic technology is used to obtain valuable information such as protein identities, expression levels and modifications (Prus et al., 2024). The study of differential protein expression in host cells between the virus-infected group and the uninfected group is helpful for analyzing functional sites, thus revealing the mechanism of virus action in the process of infection and providing research ideas for the study of targeted drugs (Kwon et al., 2021). Proteomic methods have been used to study proteomic changes in cells infected with classical swine fever virus, infectious bursal disease virus, porcine circovirus, pseudorabies virus and SARS-CoV *in vitro* (Gordon et al., 2020; Liu et al., 2020). The selected differential proteins were further screened for key proteins according to the ratio value and *P*-value, which can be used as the basis for further research. For example, the MAP3K2 protein, a key upstream kinase in the MAPK signaling pathway, can activate downstream MAP2Ks, participate in the cellular stress response and immune response, participate in tumor cell proliferation and regulate AR protein degradation (Huang et al., 2021; Gregory et al., 2001). AKT can affect its activity by phosphorylating members of the MAPK pathway, such as GSK-3, thus affecting the transduction of the MAPK signaling pathway (Hinz and Jücker, 2019). AKT1 is also related to metabolic regulation, migration and invasion of cells, which may be achieved by phosphorylation of proteins such as palladin and vimentin (Shin et al., 2002; Bae et al., 2012). The role of PKN2 in signaling pathways is related mainly to the regulation of lipid molecules such as phosphatidylinositol and diphosphatidylglycerol, which play important roles in the cell membrane (Sakaguchi et al., 2019). PKN2 has been found to regulate the AKT/mTOR pathway and promote the repair of peripheral nerve injury (Wing et al., 2020).

Currently, the mechanism by which the CSFV NS5A protein affects the MAPK and PI3K-mTOR signaling pathways remains to be further studied. CSFV NS5A lentivirus was constructed and successfully infected 3D4/21 cells. After 48 h of infection, proteomic analysis revealed 3,195 quantifiable proteins in the NS5A/control group, including 1,539 upregulated proteins and 1,656 downregulated proteins. According to the analysis data, there were 23 upregulated proteins and 16 downregulated proteins in the MAPK signaling pathway and 5 upregulated proteins and 15 downregulated proteins in the PI3K–mTOR signaling pathway. Western blotting was used to detect the expression of key proteins in the pathway, and the results revealed that with increasing CSFV NS5A infection time, the expression of IKBKG, AKT1, KRAS2, CDC37, and PKN2 decreased, whereas the expression of MAP3K7 and MAP3K2 increased.

In this study, we initially screened and identified key proteins related to NS5A in the MAPK and PI3K-mTOR signaling pathways in porcine macrophages. In subsequent experiments, we will screen the “target proteins” that interact with the NS5A protein from the abovementioned differentially expressed proteins, identify the key regions of the interaction between the NS5A protein and the “target proteins,” and explore the impact on the binding and activation of cascade proteins downstream of the “target proteins.” Moreover, we will clarify the effects of the “target proteins” on the proliferation of CSFV and the expression of the NS5A protein to further explore the molecular mechanism by which NS5A of CSFV regulates the MAPK and PI3K-mTOR signaling pathways. This study provides support for revealing the mechanism by which CSFV evades the host's antiviral immune clearance.

## 5 Conclusion

In this study, CSFV NS5A lentivirus was successfully constructed and used to infect porcine macrophages. Samples infected for 48 h were collected for proteomic analysis. A total of 3,195 differentially expressed proteins in the NS5A/control group were obtained, including 1,539 upregulated proteins and 1,656 downregulated proteins. Among them, there were 23 upregulated proteins and 16 downregulated proteins in the MAPK signaling pathway, 5 upregulated proteins and 15 downregulated proteins in the PI3K/mTOR signaling pathway, and two signaling pathway maps were drawn. In 3D4/21 cells, with increasing NS5A infection time, the protein expression of AKT1, IKBKG, CDC37, MAP3K2, and PKN2 decreased, whereas the protein expression of KRAS2 and MAP3K7 increased. Under CSFV infection conditions, the NS5A protein affects proteins in the MAPK and PI3K-mTOR signaling pathways in porcine macrophages. With increasing infection time, the expression levels of IKBKG and KRAS2 increased, whereas the expression levels of MAP3K7, MAP3K2, AKT1, CDC37, and PKN2 decreased. A corresponding key protein interaction network was constructed, laying the foundation for further exploration of the molecular mechanism by which CSFV NS5A regulates the MAPK and PI3K-mTOR signaling pathways. In the context of CSFV infection, compared with that in the uninfected state, the expression of some differentially expressed proteins exhibited the opposite trend, which provides new ideas and research directions for subsequent experiments.

## Data Availability

The original contributions presented in the study are included in the article/supplementary material, further inquiries can be directed to the corresponding authors.
